# Gut Bacterial Communities of *Dendroctonus valens* and Monoterpenes and Carbohydrates of *Pinus tabuliformis* at Different Attack Densities to Host Pines

**DOI:** 10.3389/fmicb.2018.01251

**Published:** 2018-06-14

**Authors:** Dandan Xu, Letian Xu, Fangyuan Zhou, Bo Wang, Shanshan Wang, Min Lu, Jianghua Sun

**Affiliations:** ^1^State Key Laboratory of Integrated Management of Pest Insects and Rodents, Institute of Zoology, Chinese Academy of Sciences, Beijing, China; ^2^Hubei Collaborative Innovation Center for Green Transformation of Bio-Resources, College of Life Science, Hubei University, Wuhan, China; ^3^Shandong Provincial Key Laboratory of Applied Microbiology, Ecology Institute, Qilu University of Technology (Shandong Academy of Sciences), Jinan, China; ^4^Key Laboratory of Tropical Forest Ecology, Xishuangbanna Tropical Botanical Garden, Chinese Academy of Sciences, Menglun, China; ^5^Institute of Health Sciences, Anhui University, Hefei, China

**Keywords:** *Dendroctonus valens*, gut microbiota, monoterpenes, carbohydrates, attack density

## Abstract

Insects harbor a community of gut bacteria, ranging from pathogenic to obligate mutualistic organisms. Both biotic and abiotic factors can influence species composition and structure of the insect gut bacterial communities. *Dendroctonus valens* is a destructive forest pest in China. To overcome host pine defenses, beetles mass-attack the pine to a threshold density that can exhaust pine defenses. The intensity of pine chemical defenses and carbohydrate concentrations of pines can be influenced by beetle attack, both of which are known factors that modify beetle’s gut microbiota. However, little is known to what extent variation exists in the beetle’s gut communities, and host monoterpenes and carbohydrates at different attack densities. In this study, the gut bacterial microbiota of *D. valens* at low and high attack densities were analyzed, and monoterpenes and carbohydrates in host pine phloem were assayed in parallel. The results showed that no significant changes of gut bacterial communities of the beetles and concentrations of D-glucose, D-pinitol, and D-fructose in pine phloem were found between low and high attack densities. The concentrations of α-pinene, β-pinene, limonene at high attack densities were significantly higher than those at low attack densities. Our results suggested that different attack densities of *D. valens* influence monoterpenes concentration of host pines’ phloem but have no significant impact on gut bacterial community structures of *D. valens* and carbohydrate concentration of host trees’ phloem in early attack phase. Similar gut bacterial community structures of *D. valens* between low and high attack densities might be due to the quick adaptation of gut microbiota to high monoterpenes concentrations.

## Introduction

The intestinal tract of insects is colonized by a dense microbiota composed of diverse communities ranging from pathogenic to obligate mutualistic organisms (Dillon and Dillon, 2004; Engel and Moran, 2013). The gut microbes of insects have been shown to confer advantages to the host in terms of nutrient provision (Visotto et al., 2009; Muhammad et al., 2017), suppression of pathogens and parasites (Koch and Schmid-Hempel, 2011; Engel and Moran, 2013), detoxification of xenobiotics (Genta et al., 2006; Kikuchi et al., 2012; Xu et al., 2016a), pheromone production (Dillon et al., 2002; Cao et al., 2018), regulation of immune response (Rodrigues et al., 2010; Blumberg et al., 2013), and influencing insect behavior (Sharon et al., 2010). Besides insect taxonomic position and physicochemical environment of the insect gut, host’s diet and plant defensive chemicals are well known factors that are capable of significantly influencing herbivore insects gut bacterial communities (Colman et al., 2012; Engel and Moran, 2013; Mason et al., 2015).

Bark beetles (Coleoptera: Curculionidae: Scolytinae), a group of subcortical insects that feed as larvae and adults in the phloem of trees and woody shrubs (Coulson, 1979), have caused widespread coniferous tree mortality and severe economic losses around the globe (Paine et al., 1997; Gitau et al., 2013; Sun et al., 2013). Coniferous trees can produce defensive chemicals, such as monoterpenes, in defense against bark beetle attack (Smith, 1963; Byers, 1981; Phillips and Croteau, 1999; Seybold et al., 2006). Previous studies suggested that both the intensity of host chemical defenses and carbohydrate concentration are influenced by beetles’ attack (Miller et al., 1986; Leufvén and Birgersson, 1987). Gut bacterial communities of bark beetle were shown to be less diverse than that of other insects (Vasanthakumar et al., 2006; Durand et al., 2015; Briones-Roblero et al., 2017; Hernández-García et al., 2017), which may be linked to hosts’ diet, plant defensive chemicals, and gut’s environment that exert a selective pressure on the diversity of bacterial species (Colman et al., 2012; Engel and Moran, 2013; Mason et al., 2015; Hernández-García et al., 2017). Furthermore, gut bacteria of bark beetles were reported to possess many beneficial ecological functions including degradation of defensive chemicals and nutrition provision (Boone et al., 2013; Morales-Jiménez et al., 2013; García-Fraile, 2018; Howe et al., 2018). These previous studies described a complex and also elusive interaction between gut microbiota of beetles and host pines. Although gut bacteria communities of bark beetles at different life stages have been investigated (Vasanthakumar et al., 2006; Briones-Roblero et al., 2017; Durand et al., unpublished), little is known about how gut bacterial communities of beetles vary in parallel with carbohydrates and defensive chemicals concentration changes of host pines at different attack densities.

*Dendroctonus valens* LeConte (Coleoptera, Curculionidae, Scolytinae) is a destructive pine-killing invasive pest in China, which was introduced in the early 1980s from North America and has killed more than ten million *Pinus tabuliformis* Carrière trees thus far (Yan et al., 2005; Sun et al., 2013). Pioneer beetles arrive at susceptible pine trees and attract conspecifics to the host tree (Wood, 1982; Sullivan, 2016). Beyond a critical attack density threshold, host tree defenses are exhausted (e.g., monoterpenes defense), resulting in beetle establishment (Raffa and Berryman, 1983; Guérard et al., 2000). Beetles with low attack density are unable to colonize the trees and are typically killed by host pine defenses (Hedden and Pitman, 1978; Gao et al., 2005). Gut bacteria of *D. valens* have been shown to degrade host defensive monoterpenes *in vitro* and affect carbohydrate allocation in the consumed host tissue to benefit larval development (Xu et al., 2016a; Zhou et al., 2016), both of which influence bacterial community structure. A stable gut bacterial community is important for community function (Xu et al., 2016c), however, exactly how the bacterial communities change in response to fluctuations in host carbohydrates and defensive chemical concentrations during attack at different densities of beetles remains to be determined.

The purpose of this study was to evaluate the effect of low and high attack densities on gut bacterial community structure of *D. valens*, defensive chemicals and carbohydrate concentrations of host pines. We also discussed the connection between the change in bacterial community structure and defensive monoterpenes and carbohydrate variation of host pines, which may reveal how gut bacterial communities facilitate successful attack by *D. valens*.

## Materials and Methods

### Insects and Samples

Adult beetles were collected from the Lindgren funnel traps baited with kairomone lure [(+)-α-pinene: (-)-β-pinene: (+)-3-carene=1:1:1] (99%, 98%, 97% respectively. Sigma-Aldrich, China) in the Tunlanchuan Forestry Station (N 37° 48′, E 111° 44′, average elevation 1,400 m), west of Gujiao City, Shanxi province in July 2015. Sexes of bark beetles were distinguished based on the stridulation of males (Lyon, 1958). Uninfested *P. tabuliformis* trees were cut into 50 cm lengths (diameter ≥30 cm), and both ends of the bolts were immediately dipped into melted paraffin to delay desiccation. Three evenly spaced holes (80 mm in diameter) were drilled into each bolt, and a pair of adult beetles was introduced into each of the predrilled holes. The holes were secured with wire mesh (mesh size, 2.0 mm). The bolts were checked every 24 h until each pair of beetles entered the bark. If they failed to enter, a new pair of beetles was introduced. The bolts were placed vertically in plastic boxes (40 cm in diameter, 50 cm height). The lids of plastic boxes were open to keep air flowing before beetles emerged from the bolts and were closed to collect beetles when beetles emerged from bolts. The containers were stored at room temperature/humidity throughout the rearing period.

In June 2016, we randomly collected 480 adult beetles (240 females and 240 males) that emerged from these bolts and randomly chose 8 healthy *P. tabuliformis* pines (≤100 m apart, the average diameter at breast height is 38.3 ± 1.2 cm) in the Tunlanchuan Forestry Station (N 37° 48′, E 111° 44′, average elevation 1,400 m), west of Gujiao City, Shanxi province. Four pines were set as high density group, and one hundred adult beetles (50 females and 50 males) were introduced into the main stem of each pine at 0.2–0.7 m height using the methods described above. The other four were set as low density group, and 20 adult beetles (10 females and 10 males) were introduced into each tree. After 72 h, almost all beetles have bored in the phloem with the whole body under the surface and constructed 4–6 cm length galleries, which is considered as successful colonization (Birgersson et al., 1984; Zhang et al., 2000). We then dissected the phloem tissue to collect adult beetles and excise phloem tissues around inoculation point (5 mm around the inoculation points) in each tree. Complete guts were stretched out by cutting the head and separating the abdomen from the thorax of beetles, and gut samples that missed foregut, midgut or hindgut were discard. Each gut sample (*n* = 9) was put into a 2 mL Eppendorf tube, stored at -80°C for DNA extraction. These phloem tissues (*n* = 12) were immediately frozen in liquid nitrogen for monoterpene (α-pinene, β-pinene, limonene) and carbohydrate (D-glucose, D-pinitol, and D-fructose) quantification.

### DNA Extraction, PCR, Illumina MiSeq, and Sequence Processing

DNA extraction from each adult beetle gut sample from two groups (high attack density group and low attack density group) was carried out using the TIANamp Bacteria DNA kit (TianGen, China) according to the manufacturer’s instructions. The V3-V4 region of 16S rRNA gene was amplified from the bacterial DNA by polymerase china reaction (PCR) using 16S rRNA primers 341F (5′-CCTAYGGGRBGCASCAG-3′) and 806R (5′- GGACTACHVGGGTWTCTAAT-3′) (Xu et al., 2016c). The PCR reaction mixture contained 10 ng of DNA, 1 μL of 10 μM of each primer, 2 μL of 2.5 mM dNTPs, 0.3 μL Fastpfu polymerase (Transgene, China), and 4 μL 5 × Fastpfu buffer in a 20 μL final volume. The PCR were carried out in an ABI GeneAmp^®^ 9700 thermal cycler, cycling conditions were: 95°C for 10 min; followed by 30 cycles of 95°C for 30 s, 55°C for 30 s and 72°C for 45 s; followed by the final extension at 72°C for 10 min. Each sample was amplified in three technical replicates 20 μL PCR reaction and subsequently pooled together. The final PCR products were purified on 1.5% agarose gel by electrophoresis. Sequencing was performed on an Illumina platform (Illumina MiSeq PE250).

The sequencing data were preprocessed. Sequences were assigned to samples according to specific barcodes and removed barcodes and primers. Paired-end reads were assembled with FLASH (V1.2.7^1^). High-quality data (clean reads) were acquired using the QIIME (Quantitative Insights Into Microbial Ecology) software packages (V1.9.0^2^) by filtering low-quality data with default parameters (Caporaso et al., 2010). Chimeric sequences were detected and removed using UCHIME Algorithm (Edgar et al., 2011). All effective reads from each sample were initially clustered into Operational Taxonomic Units (OTUs) of 97% sequence similarity with a UPARSE algorithm (Edgar, 2013). The most abundant sequence in each OTU was selected as the representative OTU using Greengene database^3^ and annotated by the RDP classifier algorithm implemented in QIIME under a confidence threshold of 80% (DeSantis et al., 2006; Wang et al., 2007). The raw sequence reads were obtained and deposited in the NCBI Sequence Read Archive under accession number SRR5349096 (reference: BioProject PRJNA379332).

For MiSeq data analysis, rarefaction curves were estimated using the ‘alpha_rarefaction.py’ script in QIIME to test whether the sequencing efforts adequately represented the bacterial diversity within each sample. Two richness estimators (the abundance-based coverage estimator (ACE) and a non-parametric richness estimator based on distribution of singletons and doubletons (Chao1) and two diversity indices (Shannon and Simpson index) were calculated for the samples using the ‘alpha_diversity.py’ script in QIIME. The diversity indices of two groups and the relative abundances of different genera were compared using an independent *t*-test. Non-metric multidimensional scaling (NMDS) was used to visualize the sample groupings based on Bray-Curtis similarity. Composition differences were tested using ANOSIM with 10000 permutations using PAST software, version 3.05 (Hammer et al., 2001). The representative sequences of all OTUs were used to construct neighbor-joining trees with QIIME. The phylogenetic tree together with sample sequence abundance data was used for weighted Unifrac PCoA (principal coordinate analysis) which considers both relative abundance and different branch lengths in a tree, through the online Fast Unifrac program (Hamady et al., 2010). A Permutational Multivariate Analysis of Variance based on the weighted UniFrac distance (PERMANOVA, “PermanovaG” function in the “GUniFrac” package of R) was used to test for differences in community composition between two sample groups.

### Monoterpenes Concentration in the Phloem Tissue of Trees at Two Attack Densities

The dissected phloem tissues were weighed and then ground under liquid nitrogen until a fine dry powder was obtained. Twelve phloem powder samples from each group (high attack density group, low attack density group) were extracted with hexane containing an internal standard (heptyl acetate) separately and then stored at -20°C for the chemical analysis. The most three abundant monoterpenes (α-pinene, β-pinene, Limonene) in the phloem were assayed (Leufvén and Birgersson, 1987; Xu et al., 2014).

Extracts (2 μL) were injected splitless into a gas chromatography-mass spectrometer (GC-MS: Agilent 6980N GC coupled 5973 mass selective detector) equipped with an HP5-MS capillary column (0.25 mm internal diameter × 30 m; Agilent Technologies, Inc., Palo Alto, CA, United States), and the column temperature was programmed from an initial temperature of 50°C for 1 min, then increased by 5°C/min to 100°C, by 3°C/min to 130°C, and by 20°C to 320°C and held for 2 min. Components of the extracts were identified by comparing retention times and mass spectra with authentic standards and those in the NIST02 library (Scientific Instrument Services, Inc., Ringoes, NJ, United States). Quantification was performed using an internal standard (heptyl acetate) that was added to each sample.

### Carbohydrates (D-glucose, D-pinitol, and D-fructose) Concentration in the Phloem Tissue of Trees at Two Attack Density Groups

Twelve phloem powder samples from each group, high density group, low density group, were extracted by the method described by Lisec et al. (2006) with little modification. Two hundreds mg samples and 5 mL 100% methanol were put into a 10 mL centrifuge tube and shaken for 20 min at 70°C in a thermomixer at 950 rpm. After the samples were centrifuged for 10 min at 11000 ×*g* and 350 μL supernatant with 80 μL 0.2 mg/mL ribitol as an internal quantitative standard was transferred into 350 μL 100 % methanol. With 300 μL chloroform and 600 μL dH_2_O added, the samples were vortexed for 30 s and centrifuged at 2200 ×*g* for 20 min, and 150 μL of supernatant was transferred to a new 1.5 mL centrifuge tube. After the extracts were dried in a vacuum container, 40 μL of methoxyamination reagent were added into the samples and the mixtures were shaken at 37°C for 3 h. Then 70 μL of MSTFA reagent were added into samples and the mixtures were shaken at 37°C for 30 s. The supernatant was filtered with sodium sulfate anhydrous and kept in 2 mL vials (Agilent, United States) at -20°C for chemical analysis.

Quantification analysis was carried on GC (Aglient 7890A) and FID (flame ionization detector). Chromatography conditions were as follows: injection volume 1 μL without split, helium as carrier gas at 1 mL/min constant flow mode, injector temperature 230°C, HP-5 silica capillary column (60 m × 0.25 mm × 0.25 μm). Oven temperature program was isothermal for 5 min at 70°C, followed by a 5°C per min ramp to 310°C, and holding at this temperature for 12 min. Standard carbohydrates (D-glucose, D-pinitol, and D-fructose) were also tested by GC-FID to check the retention time, by which components of extracts were identified. Quantification was performed using an internal standard (ribitol) that was added to each sample.

Prior to analysis, we tested all variables for normality with the Kolmogorov-Smirnov test and homogeneity of group variances with Levene’s test, and data were analyzed using independent-samples *t*-test. Differences between two groups were considered as significant when *P*<0.05. Data were analyzed using SPSS 12.0 (SPSS Inc., Chicago, IL, United States) and figures were drawn using Origin 8.5 (Origin Lab Corporation, Northampton, MA, United States).

## Results

### Illumina MiSeq Sequencing Data and α-Diversity Analysis

**FIGURE 1:**
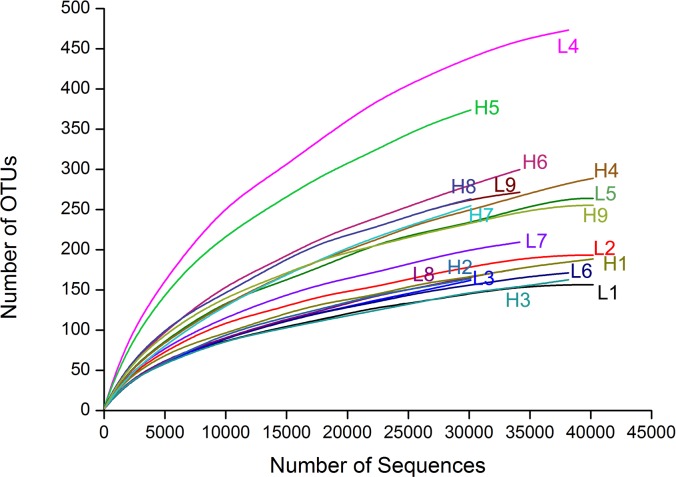
Rarefaction curves of the 18 samples based on Illumine MiSeq sequencing of bacterial communities. Color-coded lines represent different samples respectively.

In the 18 representative gut samples, we obtained a total of 681 138 sequences (90.5% of the total trimmed 752 331) and grouped into 1236 OTUs at 97% similarity cut-off level. Rarefaction curves of the 18 gut samples almost reached equilibrium, which indicated that our Illumina MiSeq analysis covered the natural bacterial diversity well (**Figure 1**). Twenty-six phyla were detected in the microbiota from 18 samples associated with *D. valens*, and of these 26 phyla, five main phyla (Proteobacteria, Firmicutes, Bacteroidetes, Actinobacteria, and Deinococcus–Thermus) representing more than 0.01% of total reads (**Supplementary Table S1**). At genus level, the sequences could be assigned to 141 genera (**Supplementary Table S1**). There were no significant differences between high density group and low density group in all five diversity indices (**Table 1**).

**Table 1:** Comparison of diversity indices (Mean ± SEM) of *Dendroctonus valens* gut bacterial community between the low and high density groups.

Index	Low density	High density
Number of OTUs	273.67 ± 55.26	248.78 ± 21.43
ACE	417.26 ± 63.77	412.43 ± 35.18
Chao1	387.52 ± 57.67	373.15 ± 30.74
Shannon diversity (H)	1.71 ± 0.17	1.76 ± 0.17
Simpson diversity	0.49 ± 0.04	0.51 ± 0.04

### β-Diversity Analysis

**FIGURE 2:**
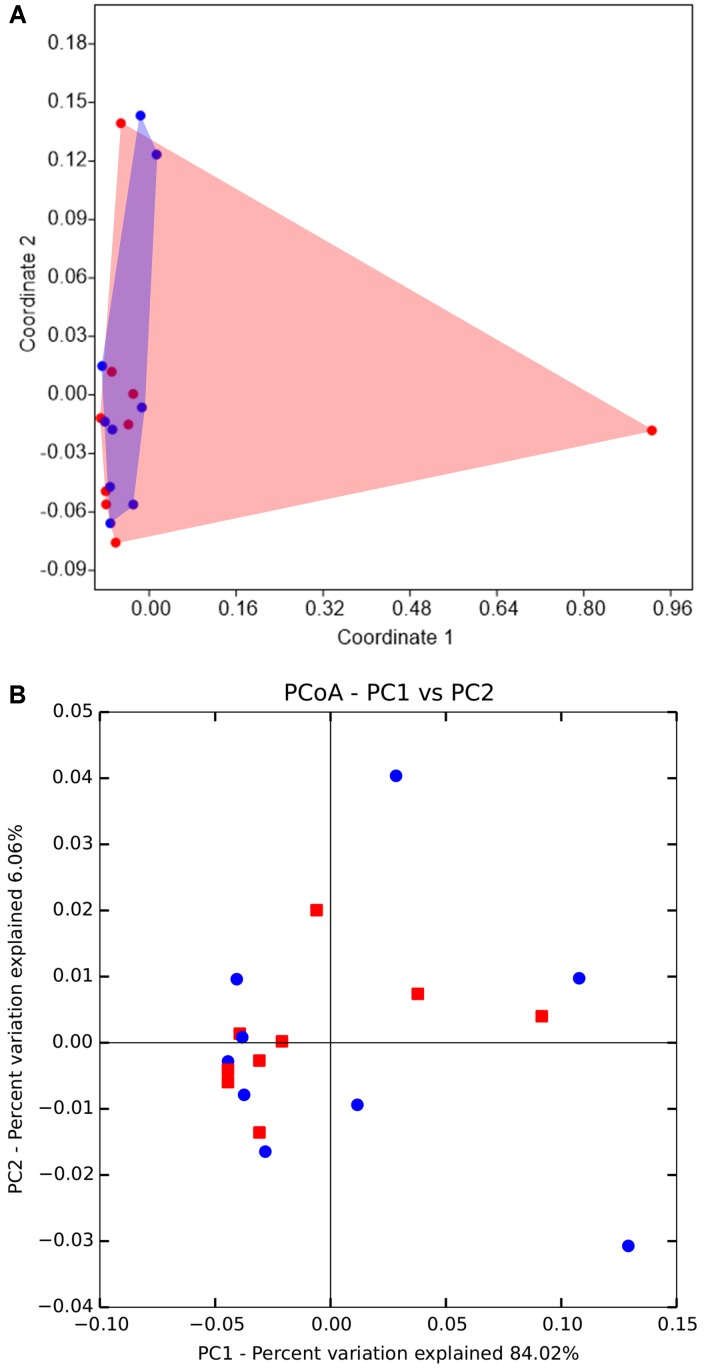
Non-metric multidimensional scaling (NMDS) and principal coordinates analysis (PCoA) of the gut microbiota of *Dendroctonus valens* between low and high attack density groups. **(A)** NMDS diagrams of 18 samples, based on Bray-Curtis distance matrix for bacterial communities that consisted of OTUs (97% similarity level). Bacterial communities of the two groups were not separated from each other. **(B)** PCoA plots based on the weighted UniFrac metric for bacterial communities. Permutational multivariate analysis of variance indicated that there were no differences between bacterial communities of the two groups. The red represents samples from high density group, and blue represents samples from low density group.

An NMDS ordination analysis based on Bray-Curtis similarities across the samples suggested that the gut bacterial communities of *D. valens* in the low attack density group were similar to those in the high attack density group (**Figure 2A**; ANOSIM, *P* = 0.54). No separation was obtained in phylogeny-based weighted UniFrac principal coordinate analysis, which was confirmed by PERMANOVA (**Figure 2B**, *P* = 0.57).

### OTU and Genus Abundance Analysis

No difference in OTUs abundance between the two groups were found (**Supplementary Table S1**). At the genus level, the genera with an abundance of at least 0.01% of the total of number of reads were present in **Supplementary Table S2**. The relative abundance of genera *Erwinia* and *Halomonas* were the highest among all genera.

### Quantification of the Monoterpenes and Carbohydrates Concentrations

Quantification of the monoterpenes in the phloem tissues results showed that α-pinene, β-pinene, limonene concentration in the phloem of trees at high attack density are 1.48 ± 0.13 mg/g, 0.19 ± 0.02 mg/g, and 0.08 ± 0.01 mg/g respectively, which are significantly higher than the concentration of the monoterpenes at the low attack density group (**Table 2**).

**Table 2:** Monoterpene quantity (mg/g) (Mean ± SEM) in *Pinus tabuliformis* phloem at low and high attack densities.

Chemical	Low attack density			High attack density				
	Concentration	Minimum	Maximum	Concentration	Minimum	Maximum	*t*	*P*
α-Pinene	0.44 ± 0.04	0.11	0.63	1.48 ± 0.13	1.08	2.47	7.65	<0.01
β-Pinene	0.07 ± 0.01	0.04	0.16	0.19 ± 0.02	0.12	0.35	4.83	<0.01
Limonene	0.04 ± 0.01	0.02	0.08	0.08 ± 0.01	0.04	0.12	5.12	<0.01

**FIGURE 3:**
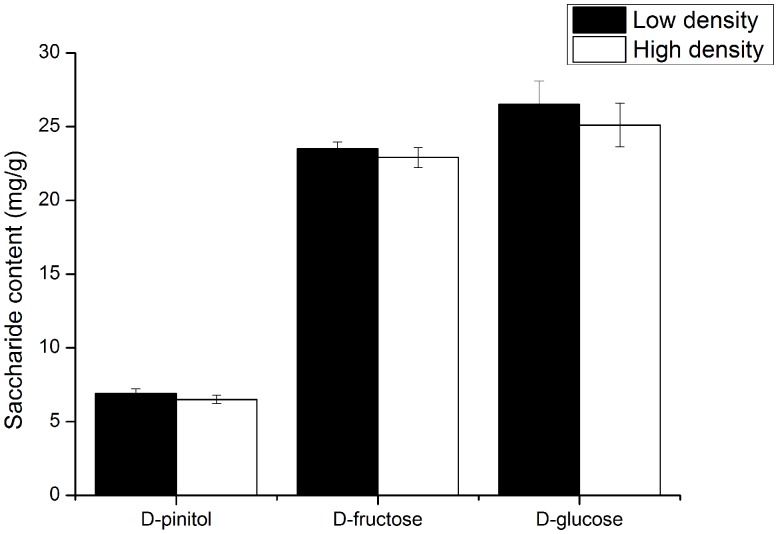
The carbohydrates concentrations (D-glucose, D-pinitol, and D-fructose) in the pine phloem tissue at low and high attack density groups. The data were analyzed using independent *t*-test.

Quantification of the carbohydrates concentration (D-glucose, D-pinitol, and D-fructose) in the phloem tissues results showed that D-glucose, D-pinitol, and D-fructose concentration in the phloem at the high density group were not significantly different from the concentrations at the low attack density group (**Figure 3**), and D-glucose is the highest abundant carbohydrate in the phloem, D-pinitol is the lowest abundant carbohydrate.

## Discussion

Our results confirmed that the different attack densities of *D. valens* can influence host defensive monoterpenes concentration. α-Pinene and β-pinene are the most abundant defensive monoterpenes for host *P. tabulaeformis* (Chen et al., 2006; Xu et al., 2014), and their concentrations rapidly accumulate after beetle attack (Miller et al., 1986; Leufvén and Birgersson, 1987). In comparison with the concentration of the monoterpenes in healthy tree (α-pinene, 0.52 ± 0.17 mg/g; β-pinene, 0.07 ± 0.02 mg/g; limonene, 0.02 ± 0.01 mg/g) (Xu et al., 2014), our results showed that monoterpenes concentrations are generally elevated by beetles’ attack in both low and high attack density groups (**Table 2**). Furthermore, we found that its concentration in the phloem at high attack density is significantly higher than those at the low attack density (**Table 2**). Plant defenses are basically regulated by genetic factors, and many abiotic factors (e.g., light, ultraviolet radiation, seasonal variation, altitudinal variation, drought) and biotic factors (e.g., phytophagous insect, pathogens, fungus) have also been shown to influence its regulation (Close and McArthur, 2002; Solar et al., 2006; Spitaler et al., 2006; Adams et al., 2008; Ballare, 2014; Wang et al., 2015). Once being attacked by insects, plants release a variety of volatiles from the insect feeding damage site, and the profile of volatiles emitted is markedly different from those undamaged plants (Paré and Tumlinson, 1999; Forkner et al., 2004). Several studies showed that defensive monoterpenes of pine trees significantly increased after attack by beetles (Miller et al., 1986; Leufvén and Birgersson, 1987; Wallin and Raffa, 1999). It was also reported that some Chinese fungal associates of *D. valens* induced higher concentration of other defensive chemicals including diterpene resin acids and naringenin in *P. tabuliformis* (Cheng et al., 2015, 2016). In the study, whether or not the associated microorganisms of *D. valens* led to the variation of monoterpenes concentration needs further study.

Although monoterpene intensity was significantly different between the low attack density group and the high attack density group, no significant differences of *D. valens* gut bacterial community structure were found (**Figure 2**). This may partly be explained by the quick adaptation of gut microbiota to high concentration of α-pinene (Xu et al., 2016c). It was reported that α-pinene can alter *D. valens* gut bacterial community structure in 6 h, but this change was recovered to the original bacterial community after 48 h (Xu et al., 2016c). Besides, diet has proved to play a major role in shaping gut bacterial communities for model insect *Drosophila melanogaster* and insects that ingest lignocellulose-derived substances (Chandler et al., 2011; Colman et al., 2012). But the carbohydrates concentration (D-glucose, D-pinitol, and D-fructose) in the diet of *D. valens* between the two groups were similar (**Figure 3**), which is independent of different attack density. Thus, whether different carbohydrates in diet would influence the gut microbiota need further study.

The most abundant genera in this study were *Erwinia* and *Halomonas* (**Supplementary Table S2**), which is not consistent with the results of gut microbiota in emerged insects which were captured in newly attacked pine stumps or fed phloem media without monoterpenes (Xu et al., 2015, 2016b). *Erwinia* are common gut bacteria in *D. valens* described in previous studies using both culture and uncultured method (Xu et al., 2015, 2016b), but *Halomonas* was first reported in *D. valens* guts. *Halomonas* have been found in other insects system, e.g., the pine wilt disease insect vectors *Monochamus* *galloprovincialis* and *M. alternatus* (Alves et al., 2016) and pine weevil (Ölander, 2013). As the environmental acquisition of diverse microbes has been shown to lead to the change of gut bacterial assemblage (Mason and Raffa, 2014), *D. valens* may acquire the *Halomonas* bacteria from host environment, which needs further study to confirm e.g., setting a negative control to explore initial conditions of microbiota of emerged beetles. In addition, *Halomonas* are able to produce cellulase and have cellulolytic activity (Huang et al., 2010; Shivanand et al., 2013). The genome analysis of *Halomonas* sp. strain KO116 indicated that several relevant genes required for lignin degradation were highly observed in KO116 genome (Kameshwar and Qin, 2016). The phloem of *P. tabuliformis* is rich in cellulose, thus, the high abundance of *Halomonas* in the gut of *D. valens* may facilitate its nutrients uptake. Besides, *D. valens* in host pines trees at both low and high attack densities have a relative stable gut bacterial community structure (**Figure 2**), which is also important for the communities to conduct ecological functions.

Our result showed D-glucose concentration in the phloem is the highest and D-pinitol is the lowest abundant carbohydrate (**Figure 3**), which is not consistent with the results executed in healthy *P. tabuliformis* (D-pinitol, 21.96 ± 4.10 mg/g; D-fructose, 14.82 ± 3.68 mg/g; D-glucose, 18.12 ± 6.65 mg/g) (Zhou et al., 2016). The phenomenon may attribute to the bacteria-fungi interactions associated with *D. valens* that regulate carbohydrate concentration in the phloem. Several dominant culturable bacteria including *Pseudomonas* associated with *D. valens* inhibited D-glucose consumption of *Ophiostoma minus* and forced *Leptographium procerum* to consume D-pinitol prior to D-glucose, thus, may lead to increase in D-glucose concentration and decrease in D-pinitol concentration (Zhou et al., 2016). Although soluble sugars and starch content in phloem were significantly changed after 72 h attack initiated by bark beetles (Miller et al., 1986), previous studies also suggested that carbohydrates contents were changed by beetles’ attack after several weeks or months (Dunn and Lorio, 1992; Wiley et al., 2016), thus 72 h after the attack of *D. valens* may not long enough to induce the variation of carbohydrates content in pine trees. In future experiments, we plan to prolong the sampling time after the attack of *D. valens* and inoculate the bacteria and fungi to the phloem directly to confirm whether the interactions contribute to the result.

## Author Contributions

The study was jointly conceived by DX, LX, FZ, BW, SW, ML, and JS. Experiments were designed by DX, LX, FZ, SW, and ML. DX, LX, FZ, and BW prepared the manuscript. LX, DX, FZ, and ML edited the manuscript. DX, LX, and SW carried out the experiments.

## Conflict of Interest Statement

The authors declare that the research was conducted in the absence of any commercial or financial relationships that could be construed as a potential conflict of interest.

## Footnotes

1 http://ccb.jhu.edu/software/FLASH/

2 http://qiime.org/index.html

3 http://greengenes.secondgenome.com/

## Supplementary Material

TABLE S1 List of OTU shared between gut microbiota of bark beetles in low and high attack density groups.

TABLE S2 Abundance of all genera in each sample. The abundance is presented in terms of percentages of the total sequences in a sample.
